# Training the next generation of plastics pollution researchers: tools, skills and career perspectives in an interdisciplinary and transdisciplinary field

**DOI:** 10.1186/s43591-023-00072-4

**Published:** 2023-11-01

**Authors:** Denise M. Mitrano, Moritz Bigalke, Andy M. Booth, Camilla Catarci Carteny, Scott Coffin, Matthias Egger, Andreas Gondikas, Thorsten Hüffer, Albert A. Koelmans, Elma Lahive, Karin Mattsson, Stephanie Reynaud, Stephan Wagner

**Affiliations:** 1 Department of Environmental Systems Science, ETH Zurich, Universitätstrasse 16, 8092 Zurich, Switzerland; 2 Institute of Applied Geoscience, Technical University of Darmstadt, Schnittspahnstrasse 9, 64287 Darmstadt, Germany; 3 SINTEF Ocean, Brattørkaia 17C, 7010 Trondheim, Norway; 4 Plastics Europe, Rue Belliard 40, Box 16, 1040 Brussels, Belgium; 5 California State Water Resources Control Board, 1001 I St., Sacramento, CA 95605 USA; 6 The Ocean Cleanup, Coolsingel 6, Rotterdam, 3011 AD The Netherlands; 7 Egger Research and Consulting, Ullmannstrasse 13a, 9014 St. Gallen, Switzerland; 8 Department of Geology and Geoenvironment, University of Athens, Athens, Greece; 9 Department of Environmental Geosciences, University of Vienna, Josef-Holaubek-Platz 2, 1090 Vienna, Austria; 10 Aquatic Ecology and Water Quality Management Group, Wageningen University, PO Box 47, Wageningen, 6700 DD the Netherlands; 11 UK Centre for Ecology and Hydrology, Maclean Building, Benson Lane, Crowmarsh Gifford, OX10 8BB UK; 12 Department of Marine Science, University of Gothenburg, Fiskebäckskil, Sweden; 13 Universite de Pau et des Pays de l’Adour, E2S UPPA, CNRS, IPREM, UMR 5254 Pau, France; 14 Institute for Analytical Research, Hochschule Fresenius, Limburgerstrasse 2, 65510 Idstein, Germany

**Keywords:** Plastic, Student, Early career, Training, Interdisciplinary, Transdisciplinary

## Abstract

Plastics pollution research attracts scientists from diverse disciplines. Many Early Career Researchers (ECRs) are drawn to this field to investigate and subsequently mitigate the negative impacts of plastics. Solving the multi-faceted plastic problem will always require breakthroughs across all levels of science disciplinarity, which supports interdisciplinary discoveries and underpins transdisciplinary solutions. In this context, ECRs have the opportunity to work across scientific discipline boundaries and connect with different stakeholders, including industry, policymakers and the public. To fully realize their potential, ECRs need to develop strong communication and project management skills to be able to effectively interface with academic peers and non-academic stakeholders. At the end of their formal education, many ECRs will choose to leave academia and pursue a career in private industry, government, research institutes or non-governmental organizations (NGOs). Here we give perspectives on how ECRs can develop the skills to tackle the challenges and opportunities of this transdisciplinary research field and how these skills can be transferred to different working sectors. We also explore how advisors can support an ECRs’ growth through inclusive leadership and coaching. We further consider the roles each party may play in developing ECRs into mature scientists by helping them build a strong foundation, while also critically assessing problems in an interdisciplinary and transdisciplinary context. We hope these concepts can be useful in fostering the development of the next generation of plastics pollution researchers so they can address this global challenge more effectively.

**Graphical Abstract:**

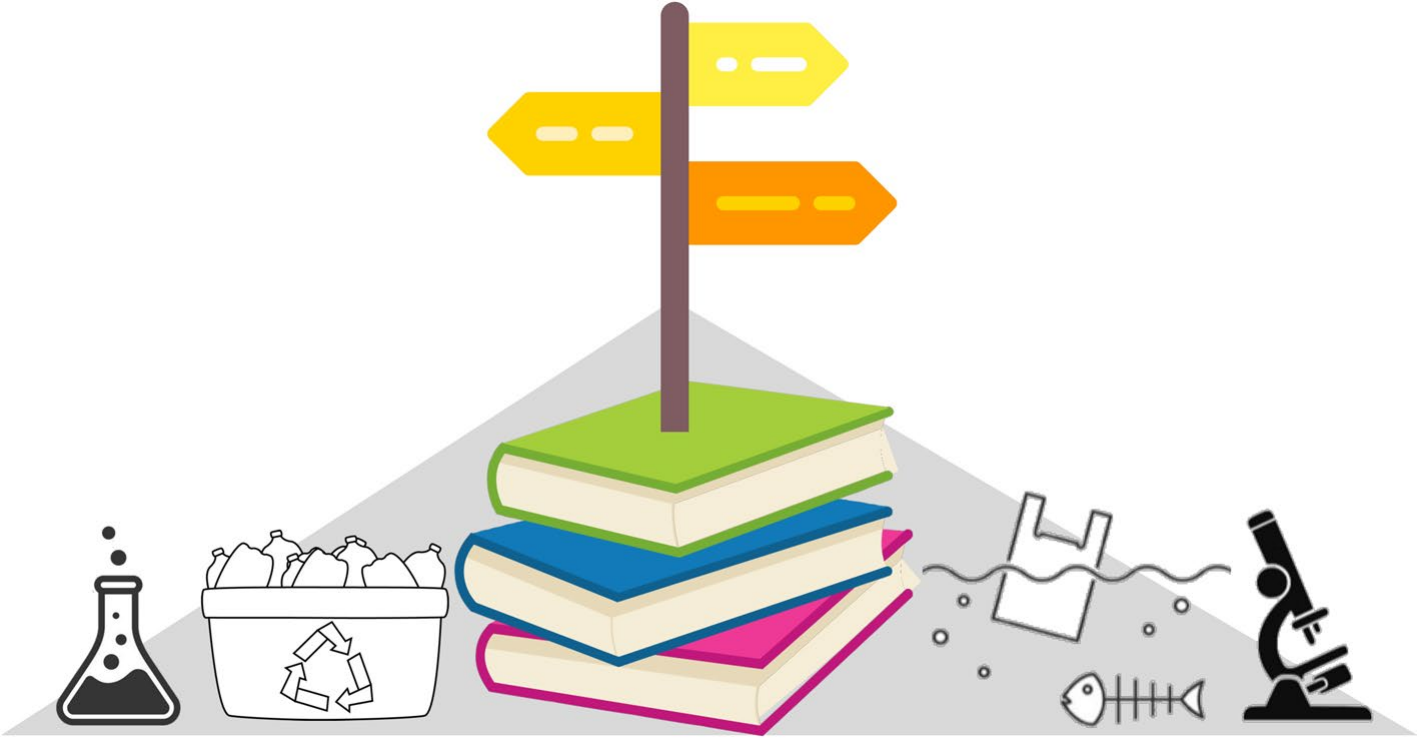

## Synopsis

Plastics pollution is a wicked problem that will require stakeholders from many different backgrounds to solve. In this perspective, we highlight how early career researchers (ECRs) can translate and appreciate their work in the context of interdisciplinary research and transdisciplinary solutions, and considerations needed for successful career development and advancement in different sectors. We also comment on how advisors can support ECRs in developing their career and create working environments which are conducive to helping students acquire the mentality and skillsets for solving globalized problems.

## Introduction

Plastics pollution, including nano- and microplastics, is anticipated to negatively impact environmental quality, human health and affect ecosystem services through interactions with biota and altering biogeochemical cycles. An increasing number of researchers have been studying the occurrence and effects of (nano- and micro)plastics pollution in various contexts [1]. Because of the complex nature of such a large and multi-faceted topic, plastics pollution research attracts scientists from diverse disciplines, ranging from polymer chemists to environmental scientists to human and ecotoxicologists to social scientists (to name a few). Like other wicked problems, plastic pollution defies simplistic solutions, is challenging to frame, involves conflicting values and interests [2], and is riddled with uncertainties [3, 4]. This makes plastics pollution a societal, economic and environmental challenge which needs interdisciplinary research and transdisciplinary solutions since the successful management, prevention and mitigation measures need to integrate knowledge across various academic disciplines and non-academic stakeholders. Creating an environment for interdisciplinary and transdisciplinary research will facilitate finding solutions across traditional disciplinary boundaries. Fostering dialogues, mutual learning, and knowledge co-production across disciplines will accelerate and diversify research and ensure challenges along the entire plastics lifecycle are addressed. This integrative approach links scientific innovation with solutions to societal problems, while still acknowledging and accepting local contexts and uncertainty in knowledge [5]. Although interdisciplinarity is a key feature of transdisciplinary research, transdisciplinarity goes beyond interdisciplinarity by adding cooperation between science and society to the inner-scientific cooperation between different disciplines [6, 7].

Academic research is often criticized as scientists working in silos. Traditionally, embarking on a PhD has meant focusing on developing knowledge and skills in a narrow field of research on a stand-alone topic. While this typically requires dialogue with other experts within a given field, ECRs can be isolated from other disciplines. This siloing makes communication across disciplines more challenging and may limit the scope of research questions individual researchers tackle. There is, therefore, a growing need for today's ECRs to have awareness of different scientific disciplines and engagement with a wide variety of stakeholders in the area of plastics pollution to both better contextualize their work and to deliver results that contribute to meaningfully minimizing the impacts of plastics throughout their entire life cycle. Indeed, many ECRs are increasingly interested in seeing their work being applied to policy, technological solutions, or informing citizens. As a scientific community, we need to cultivate collaborative research approaches, and one of the most important ways to do this is by providing ECRs with the opportunity to develop skill sets that allow them to do this effectively across a broad range of different career paths after their formal training ends.

There are both opportunities and challenges for ECRs, who must simultaneously grow their scientific expertise while also developing effective communication and project management skills at the start of their career. As for all scientists, ECRs working in plastics pollution research must build a strong foundation in the scientific method, which includes developing important hypotheses and objectives, designing laboratory experiments and field campaigns, technical skill sets to analyze samples and data, modeling, and interpreting and reporting these appropriately. Development of these tools is critical to the success of ECRs being able to answer key questions in the field and to help build the foundation of scientific knowledge about the plastics lifecycle and impacts. To communicate their results, ECRs must consider the broad audience(s) that are interested in their scientific results, including peers in plastics pollution research, other scientists in related fields, industry, policy makers and the public. To make their research applicable, ECRs should be able to contextualize their own research, envisage how it can be utilized beyond their own principal domain, and be able to critically evaluate plastics pollution related research from other disciplines [8]. Ultimately, understanding and respecting the positions of other (academic and non-academic) interest groups and appreciating that multiple points of view must be considered when tackling plastics pollution is a necessity. Regardless of whether an ECR pursues an academic career path or not, the holistic development of these aspects provides a more broadly applicable and transferable skill set that includes evaluation of complex information, assessing contradictory evidence and effective communication. With respect to inter- and transdisciplinary research in particular, further expertise spanning the traditional boundaries of research and administrative/managerial tasks are necessary, including cross-boundary communication, project leadership and management, research approaches, administration, and outreach to a variety of stakeholders [9, 10]. More specifically, integral competencies include 1) translational skills between diverse groups of scientists and other stakeholders, 2) effective knowledge synthesis between disciplines, and 3) balancing intellectual leadership with administrative responsibilities between disciplines.

In June 2022, the authors of this perspective, all of whom are senior researchers and/or actors in the field of plastics pollution from various disciplines and career paths, organized a workshop aimed at stimulating broader thinking and communication skills for plastics pollution ECRs. The workshop format coupled scientific expertise in plastics pollution research and targeted skills development, collectively allowing ECRs to embrace the challenges and opportunities of working in this field. In this perspective, we aim to provide a framework for coaching and training ECRs working on complex societal problems, such as plastics pollution. Here plastics pollution is used as an exemplary case, but the concepts would also be applicable to ECRs in other inter- and transdisciplinary fields. We hope these concepts can foster the development of the next generation of plastics pollution researchers. Our goal is for this perspective to prove useful for both ECRs who are building their careers, as well as more established researchers who want to help ECRs achieve more holistic profiles to tackle multi-faceted global challenges. While we do not mean to imply that all ECRs will or should develop a profile which contains inter- and transdisciplinary aspects, we would like to stress that having an awareness of how ones’ work fits into a larger picture is advantageous. We explore how to best help ECRs grow into mature scientists by building a strong foundation while also critically assessing problems in an interdisciplinary and transdisciplinary context. Consequently, we hope this perspective can act as a catalyst for reflection on the diversity of approaches when developing ones’ skillsets and scientific approach, independent of whether an ECR chooses to work within or outside academia.

## Challenges and opportunities as an ECR in the plastics research field

There is currently widespread acknowledgement of the challenges posed by plastics pollution, including strong public awareness and interest in addressing the problem, and consequently plastics pollution research has increased significantly in recent years. Many governments, regulatory bodies and research commissions have supported large, well-funded initiatives to address the issue, consequently providing ECRs with a diversity of academic backgrounds the opportunity to delve into plastics pollution research. The recognized need to address multiple drivers (e.g., environmental, economic, political, societal, etc.) has generated a unique opportunity for researchers to be part of a global effort to improve our understanding on this timely issue [3]. This is in line with a general increase in the proportion of transdisciplinary projects that aim to grasp the complexity of real-world problems and sustainability challenges [11]. Consequently, plastics pollution ECRs are far from being alone in gaining their scientific qualifications outside of disciplinary lines.

**Fig. 1:**
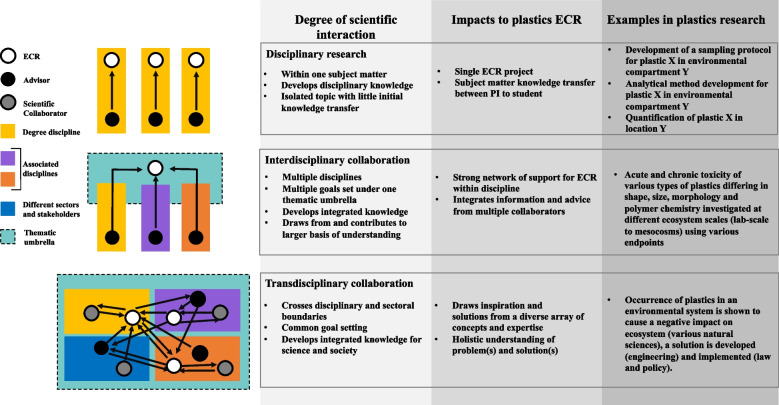
Solving the plastic problem will always require breakthroughs on all levels of science disciplinarity. At each level, there are different degrees of interaction between individuals and an ECRs placement in the working environment. Examples of different research questions, approaches and expected outcomes from the plastics pollution field are provided for each case. Adapted from Morton et al. [12]

Solving the plastic problem will require breakthroughs at all levels of research disciplinarity. Some of these will be mono-, multi-, or interdisciplinary research and these can support interdisciplinary discoveries and underpin transdisciplinary solutions (Fig. 1) [12]. In contrast to the traditional model where ECRs focus on a narrowly defined topic in a given field, the breadth of information continually generated on this topic and the complexity of the problem requires researchers to be well-informed across a range of disciplines. Any researcher that embraces this complexity has the opportunity to build a strong and diverse scientific network to tackle concepts and projects that require input across traditional disciplinary lines [13]. Working in this way often requires more exploration of approaches and methodologies than disciplinary research, which can necessitate learning additional skillsets. A benefit for ECRs is that they can have several expert mentors to increase their technical skills. Although individual ECRs will not be experts in all fields associated with a complex study, they may be able to complement the skillsets and viewpoints of others to have an appreciation and integration of research from other disciplines. Consequently, they will have a broader, more holistic view of the plastics pollution issue and can translate this way of higher-level thinking in contextualizing their work.

ECRs need opportunities to develop research planning and management skills, and Djinlev et. al have summarized 10 challenges specific to ECRs conducting inter- and transdisciplinary research [14]. Creating their project framework will often require collecting, sorting, and prioritizing ideas from different colleagues and sources to develop a cohesive plan [15], where they can face conflicting methodological standards and lack of integration across knowledge types. When in theory an ECRs’ project involves multiple experts from different domains and they can synthesize different types of information to draw more robust and overarching conclusions, in practice this necessitates an ECR to both build their scientific foundation and contextualize their findings simultaneously. Coordinating meetings, updates and dissemination activities across project partners also allows ECRs to develop project management skills, yet this often comes with a significant investment of time. Indeed, ECRs involved in inter- and transdisciplinary research often cite work overload as a significant downside. Consequently, coordinating different research lines within a project often takes more time than in disciplinary research, which can ultimately decrease the output of peer-reviewed journal articles—traditionally a key metric for academic success. ECRs, therefore, need to perform a balancing act to remain up to date with literature and development of research concepts in contributing disciplines, while at the same time staying focused on the objectives of their own research project. Efficiency with time management through making goals and targets on a realistic time schedule is paramount. Conversely, academia in some countries is moving towards “science for societal impact” rather than impact as measured by bibliometric statistics, benefiting said balance [16–18]. Nevertheless, deciding on where to spend time, effort, and focus is a delicate and often stressful decision-making process for ECRs, where some worry if the scientific rigor of transdisciplinary research projects is comparable to discipline-specific projects in the context of a PhD program [14, 19].

**Fig. 2:**
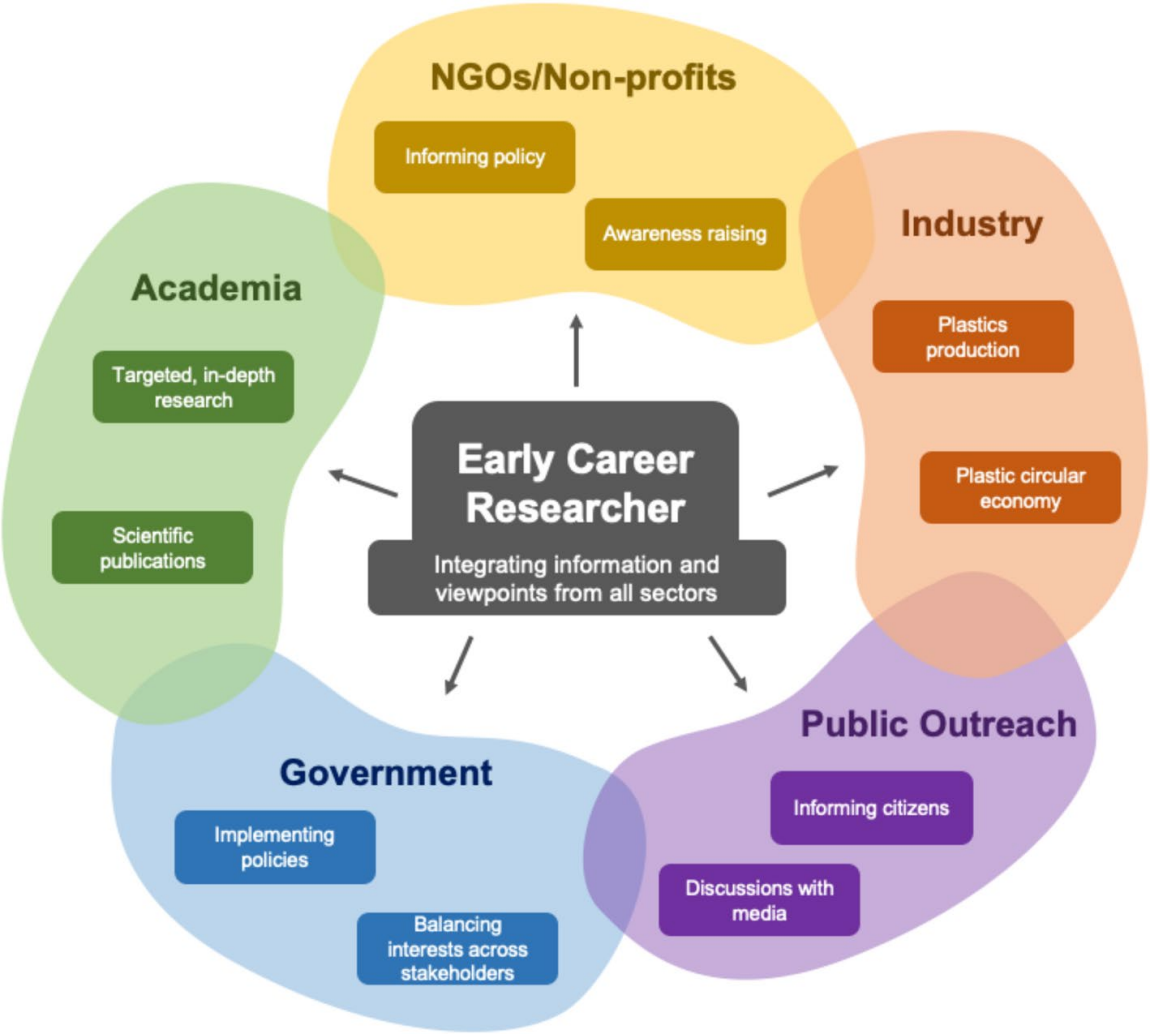
In transdisciplinary research, each stakeholder will have different expertise, value prioritization and expected or ideal outcomes. For ECRs working in the field of plastics pollution research, understanding these different viewpoints and learning how to effectively communicate with academic and non-academic stakeholders will both help them contextualize their current research project and work in a more efficient and collaborative manner. Colored boxes show examples of tasks and challenges in the respective sector

Increasingly, plastics pollution research extends beyond the academic realm to provide scientific evidence to support policy decisions and inform the public [20]. Open discussion with these stakeholders is where transdisciplinarity starts in earnest (Fig. 2). ECRs can play a pivotal role in strengthening cooperation between the scientific community and civil society in particular, but they can also make connections between industry and policy stakeholders as well. This exciting opportunity for ECRs to communicate their work goes hand in hand with the responsibility of clearly acknowledging the limitations of their study to avoid overinterpretation of results [21, 22]. ECRs can utilize established platforms to communicate with both peers and stakeholders through a variety of routes, ranging from peer-reviewed scientific publications to simplified public media outlets that is easier for non-scientists to comprehend. Open access publishing offers a system that facilitates sharing of scientific discourse to an audience beyond academia, e.g., journalists, policy makers, government, industry, and the public [23]. Aside from peer-reviewed manuscripts, the importance of sharing data openly will advance the state of the field [24]. Additionally, ECRs can directly communicate to an information-thirsty global community through social media, but facts need to be delivered in targeted and concise ways [4, 25]. All scientists, including ECRs, should consider the scientific level of their target audience when using social media to avoid claims that may be exaggerated and misinterpreted [25]. Developing the skills early in ones career to communicate effectively to different audiences can significantly boost an ECRs’ job perspectives.

## Developing skills and expertise for transdisciplinary research

The concepts of transdisciplinary research will not replace disciplinary research topics, but there are many complementary aspects. In the context of plastics pollution research, for example, we still need efforts which focus on assessing hazards of different materials. However, this disciplinary research will only reach its full impact when it is combined with an exposure assessment and further integrated into risk assessment, especially for more environmentally relevant scenarios. Eventually, environmental regulations could be passed based on the collective results across all these aspects. Experimental planning, communication and application of results must be coordinated to reach more impactful conclusions. For this, common vocabularies are needed to overcome barriers (e.g., dialects, nomenclature, data classification, etc.) that occur across and even within disciplines [26]. Addressing the complex questions about plastics pollution increasingly requires the ability to work and communicate across disciplines and sectors in order to gain a perspective that encompasses the multi-level and multi-faceted conditions involved with this issue [24, 27–29].

Often ECRs focus heavily on discipline-specific technical skills during their training, which can lay the foundation for their work. In our opinion, importance should also be placed on developing skills to synthesize and contextualize results in a broader context. Effective communication, both written and oral, is key when working in inter- and transdisciplinary research since ECRs will need to discuss with researchers and stakeholders from different backgrounds and offer access to knowledge that is not traditionally part of ones’ main discipline (Fig. 1). In this field of research, ECRs also have the opportunity to work beyond the academic setting and begin to integrate the perspectives, values, and interests of societal actors involved across the entire plastics value chain including industry, consumers, and government stakeholders. Here, the guiding question for developing skills and expertise for transdisciplinary research is: How can we learn to work in systemic ways with multiple approaches to create transformative change to avoid plastics pollution, mitigate negative impacts, and move towards sustainability? Most importantly, a good advisor for ECRs remains open minded to new ideas and has a track record in successfully guiding ECRs in completing their projects and advancing their careers. Additional attributes may include having a strong publication record themself and being active in the chosen research field, as this will allow ECRs to more easily develop their network and stay current and timely in their research objectives.

Our microplastic workshop accounted for these aspects in its agenda by aiming to train ECRs by increasing awareness of being open minded and the importance of these transferable skills in developing ones’ career. For several reasons, many scientists tend to feel that they need to know everything and revealing lack of knowledge (even if outside ones’ own research scope) is often seen as a weakness. Consequently, many ECRs may be less inclined to include concepts from other research disciplines or stakeholder viewpoints into their own work for fear of appearing less capable. We highlighted that learning how to ask for (as well as provide) clarifications from peers in other fields of research and stakeholders with different backgrounds is key to creating effective communication and reaching common goals. Creating simple, well-organized, and targeted take home messages allows others to understand and use the results of one’s work in practice. These approaches will eventually lead to increased understanding across the entire plastics pollution research community and create bridges for research which takes into account multiple viewpoints.

## Career opportunities for ECRs in the field of plastics pollution research: within and outside the academic environment

ECRs are at a stage when they must decide the future direction of their careers, and they often initially consider a career in academia as the “default” option and the best way to stay involved in cutting edge research science. Remaining in an academic setting comes with a comfortable familiarity for many, since the working environment and structures are already known, and because ECRs advisors and mentors can often more easily guide them in this direction. Nevertheless, many ECRs are fully aware of career insecurity in both the short- and long-terms and the competitive culture of “publish or perish” which can create adverse incentives within the academic research environment [30, 31]. Knowledge about the demands, and the pros and cons, of an academic career compared to other career possibilities is a clear asset for informed decision making. Here we highlight career opportunities both within and outside academia and briefly discuss their benefits and challenges. Note that the authors primarily have experience working within Europe and the US, and thus the options presented here mainly reflect these regions, although they may be applicable elsewhere. Before leaving academia, it is important to consider the potential difficulty of re-entry. Personal research interests can generally still be pursued in other sectors if they are within the general scope of the organization. However, key parameters that can limit a return to academia after working in other sectors are the opportunities to publish in peer-reviewed journals or demonstrate a track record of funding acquisition. It is also important to note that ECRs do not necessarily need to work directly in the same discipline as their graduate research. The authors of this perspective represent plastics pollution researchers employed across a range of different sectors (academia, government, private sector, research institute, NGO), allowing them to provide specific insight into the different career paths available to ECRs working within this research field.

### Academia

Beyond the considerations listed above, many of which an ECR may already be aware of through their own experience and discussions with peers and advisors, it is important for ECRs to recognize that positions within academia can also vary greatly. This can include, for example, different legal framework conditions for scientists in different countries, universities which are more teaching focused versus research focused, different department structures, and the different institutional demands that vary from university to university. While in some academic departments, discipline-specific expertise (and to some extent research) is sometimes favored because of the teaching demands associated with a given professorship position [32], there is also increasing appreciation of inter- and transdisciplinary research within academic settings. That being said, transdisciplinary chairs and professorships are still rare, and a transdisciplinary track record is usually not sufficient when applying for disciplinary full professorships (which also holds true for interdisciplinary academic career paths) [19]. Facing these types of structural challenges may be even more daunting for those ECRs who want to incorporate inter- or transdisciplinary research into their portfolios, where they need to still be seen as subject matter experts and develop their academic identity [33]. While there have been calls to further establish permanent roles of those who can facilitate inter- and transdisciplinary research [34], including the development of course structures to include these concepts [35], in the short-term such institutional challenges can be an obstacle for some. Despite this, there are an increasing number of funding mechanisms which specifically support or request inter- and transdisciplinary research approaches, where the direct involvement of industry partners and other stakeholders is integral for securing the grant and for the subsequent success of the project. In recent years, there have also been initiatives developed to support transdisciplinary research, such as the *tdAcademy* network and the *td-net*, as well as those specifically targeted towards ECRs [14], such as the *International Transdisciplinary Alliance*.

### Government

A significant benefit of working in government is a predictable, stable, structured lifestyle. Many government positions often follow standard 40-h working weeks, allowing for healthy work-life balance and family-centric lifestyles that may be challenging to maintain in other sectors. Such positions typically include good benefits and pension packages, and provide unique opportunities to apply science to policy, thus more directly affecting societal change. Furthermore, scientific employees in government enjoy freedom from a number of time-consuming, potentially stressful tasks prevalent in other sectors, such as grant applications, client relations, increasing company profits, or teaching classes. In contrast, the highly structured nature of government work results in slower progress and actions hampered by complicated bureaucracies. In general, the focus of government agencies on applied science means many positions do not involve research and may comprise more repetitive work with fewer opportunities for individual creativity. However, working in a government position may allow the ECRs in the field of plastics pollution to contribute and participate in decision making processes for substantial societal changes in the field of circular economy, environmental policy and regulation, and beyond. Nonetheless, positions in the government often provide unique and valuable perspectives that can lead to numerous and exciting opportunities in further career development if one wishes to change sectors later in their career (e.g., consulting and industry sectors often value applicants with prior government experience). To this end, government employees often have opportunities to build networks with diverse stakeholders across sectors, providing high mobility for career changes.

### Private sector (industry, consultancy)

Many ECRs may not be aware of the extent of options available in the private sector (industry, trade associations, and consultancy firms), which can provide a powerful drive for change in the ongoing challenge to reach sustainability and circularity within socioeconomic systems. However, the transdisciplinarity of environmental plastics researchers is a key asset for such a transition. A scientist’s role within this sector can span from research and innovation (e.g., innovative materials, circular production processes, product safety) to strategy and solutions (e.g., policy analysis and drafting, life cycle assessment, product stewardship). An advantage of the private sector can be faster career advancement compared to academia, which has a positive effect on salary, responsibility and experience. When working within the private sector, publications do not represent an essential performance metric, relieving the constant pressure many academics experience [36]. This is connected to personal impact, where academics are typically recognised based on individual contributions and private sector researchers are seen as members of a wider organisation. As such, the private sector has much less focus on individuals achieving results and less pressure to constantly establish new investigation lines and research areas. Tight timelines and prioritisation are driven by product or business goals, which typically sees private sector researchers working in teams, integrating science and business-focused problem solving.

### Research institutes

Research institutes, whether public or private, can cover either applied or fundamental research, often providing employees an opportunity to work on a diverse range of research topics and societal challenges. Applied research should lead to the development of new knowledge, technologies and commercialization, often in close collaboration with industry partners. Research institutes are often viewed as knowledge centers, providing expert advice that informs public debate and policymaking. Scientific publishing is also important within this sector and provides a crucial platform for marketing competence. Researchers at these organizations are encouraged to follow personal interests as long as funding can be secured and it falls within the research institutes strategic scientific goals. Collaboration across the often-diverse departments and groups is encouraged, allowing researchers to participate in highly transdisciplinary projects. Indeed, research institutes can sometimes have more capacity to be responsive in redirecting resources from different expertise towards addressing urgent knowledge gaps or problems highlighted by for example, policy makers or industry. Strong links to both universities and industry provide opportunities to collaborate across sectors. A major challenge in this sector is the need to secure external funding, which can limit research freedom, place financial constraints on the scope of research, and create a barrier to exploiting new ideas quickly. There are many examples of research institutes with different mission statements, directives and funding structures, and some may have similar attributes to an academic position (e.g., advising ECRs, applying for funding, and a focus on publishing results). Conversely, others may focus more on writing white papers and disseminating information, often to the public, where production of primary research is less in focus. While it is challenging to make overarching statements with relevance to all cases and individuals, ECRs may be able to find a plethora of different roles when looking for employment opportunities within this sector.

### Non-profit and NGOs

Scientists in the non-profit and NGO sector often work at the intersection between academia, government, research institutes and the private sector, often focusing on conducting research and communicating the results to a wide(r) audience, including other scientists, industry, the public and policy makers. Talking to diverse stakeholders requires a good understanding of the broader implications of one’s research and being able to explain complex problems to non-experts. While scientists in this sector still publish peer-reviewed articles, the publication rate is likely lower than in the academic sector. Working together with non-scientists in the same organization provides opportunities to learn about other fields and skills often neglected in academia, including business development, accounting, legal, public relations and media. The sometimes-limited freedom of pursuing one’s individual research interest and the relatively lower wages compared to other sectors [37] are often considered the main downsides. Research teams can be relatively small and access to laboratory equipment limited, requiring scientists to establish collaborations with research institutes and universities. Networking through participating in international workshops and conferences is therefore typically considered essential. 

## Helping ECRs navigate their future

**Fig. 3:**
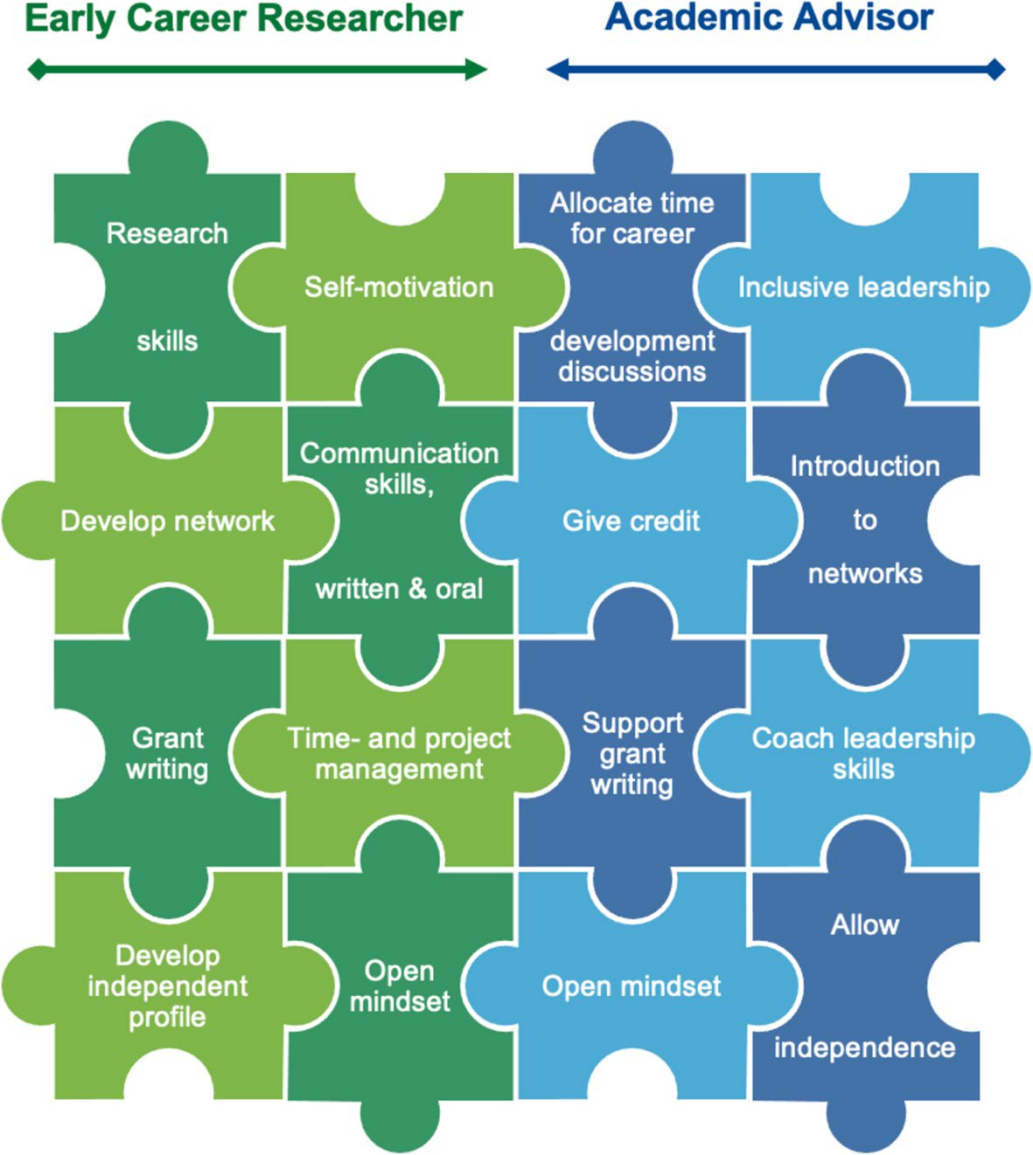
Career development of an ECR takes a team effort between the young scientist and the academic advisor. There are several key pieces to the puzzle of how to build a successful working relationship, no matter in which sector the ECR chooses to work in in the future

Advisors of ECRs play a critical role in fostering their development in both direct and indirect ways [38, 39]. This is the case regardless of the type of research the ECR is involved in, i.e., independent of if the project is disciplinary, interdisciplinary or transdisciplinary. Forward-thinking ECRs are often eager to develop transferable skills, explore career options and discuss important life decisions, but sometimes lack sufficient mentorship either because their advisor is less experienced or because they place more weight on scientific output due to personal expectations and heavy workloads. However, advisors should dedicate time to discuss personal development and employment options with ECRs, both in individual and research group settings, as well as be open to discussing non-academically focused career choices. The ECR should also play an active role in developing their research profile, improving their communication skills and building their professional network. Young researchers are often adept at finding ways to strengthen research-related competencies, but they should also actively seek out opportunities to improve soft skills and project management capabilities and take career readiness courses (e.g., CV and cover letter writing courses, public speaking courses, etc.) to prepare themselves for their next role. Ideally, advisors should be open to the ECR spending time on these “non-academic” activities to proactively support the ECRs holistic development for future success. There are some universally applicable approaches to help ECRs navigate their future, despite the fact that each ECRs situation is unique to the individual characteristics and personality traits of the advisor and ECR, as well as the research group culture and context as a whole. Some foundational strategies of a productive working relationship from the viewpoint of both the advisor and ECR in the academic context are outlined below and in Fig. 3.

There is an unfortunate line of thinking prevalent in research settings that successful scientists are not good people managers. While it is true that until recently there has not been much dedicated training within the academic environment on effective leadership strategies, universities have begun to allocate more resources in training faculty to not only excel in research but also to better manage their teams [40, 41]. Inclusive leadership can positively impact team working dynamics by creating an environment where all employees feel respected, valued and able to contribute their best [42]. These leadership traits include such mindsets as self-awareness, curiosity, courage, vulnerability and empathy. By adopting inclusive leadership, which translates well in the context of inter- and transdisciplinary science where one has to be open to new ideas outside ones’ domain, faculty can create a shared vision within their team to manage and lead a heterogenous group of people, while respecting their individuality and promoting personal growth. Modern academic systems put significant pressure on faculty to simultaneously produce world-class research, teach, acquire external funding, provide services to the research field (e.g., editorial and reviewing duties) and administrative tasks, resulting in a very demanding workload. Consequently, individual and personalized development of ECRs may suffer due to lack of time and/or additional responsibilities may be placed on ECRs to alleviate pressure from the advisor. Nevertheless, it is important that a good ECR mentor is motivated to allocate time to supervision and has a genuine interest in the projects and careers of the ECRs. The additional responsibilities taken on by the ECRs should also be officially acknowledged and recognized, so that ECRs do not remain in the shadow of their academic advisor.

Advisors can also take more concrete measures to support the ECRs working with them, especially in the context of fostering research and independence, developing project management skills and introductions into professional networks. Over time, ECRs will develop their own ideas but designing research plans takes patience and practice. ECRs should be coached to develop their own subprojects, which could include supervision of younger researchers (e.g., Masters students, guest researchers) and/or writing their first grant applications, as appropriate for their setting. While larger grant applications are often not applicable for ECRs because of limited employment contracts, there are many funding opportunities specifically targeted for ECR career development and mobility through national- and international funding programs (e.g., Marie Skłodowska-Curie Actions, Fulbright, and Humboldt research fellowships) or directly at the intended host university. Such applications allow an ECR to learn how to write a compelling research narrative, plan project timelines and budgets and develop independence, even if ultimately the grant is not funded. An easy way to help ECRs further develop their professional network is by involving them in meetings with stakeholders in projects, administration, and media outlets, which helps them foster contacts and experience. They can also be supportive of the ECR visiting workshops and conferences with a mixed audience (i.e., not only academically focused) to help broaden the ECRs’ field of view for them to assess future employment opportunities. Creating an environment that allows an ECR to develop their own ideas of their future career, developing competences and networks and offering freedom for their own ideas and projects will improve their chances of success.

ECRs ultimately must take planning their futures into their own hands, which requires a significant amount of self-motivation, self-efficacy and resilience. For more senior ECRs, gaining autonomy is key in order to develop one’s own research profile and expertise in order to be viewed as an independent expert – especially when remaining in academia. In essence, the ECR needs to create visibility for themselves and their work by 1) differentiating their research from the core expertise of their advisor, 2) building a network for future collaborations and job prospects and 3) having a broader understanding of the field to participate in transdisciplinary research activities or be competitive in a non-academic setting. For their future career, ECRs require not only expert knowledge and skills, but also general competencies in time- and project management and communication skills to different audiences and stakeholders. The theoretical base of these competences can often be learned in graduate schools, summer schools and voluntary courses in most academic institutions, but should also be applied and developed in practice. To do so, peer-to-peer exchange and training and being involved in broader project contexts, where ECRs have the possibility to communicate with a variety of different stakeholders should be stimulated. The active investment in building up one’s own network, getting involved in the networks of the academic advisor or other group members is key to a career in academia but also in other sectors. To build one’s own network, ECRs should attend conferences and workshops as an active participant: physical presence alone often does not lead to relationship building opportunities. Being involved in different projects with many participants and investing time to socialize with colleagues and stakeholders will build trust in their network. This network will help one know about new calls for research projects, invitations to take part in projects, provide support with expertise one does not have, or advertise ECRs skills to colleagues and future employers. Taking part in broader academic discussions (e.g., ethics, responsibilities of science), public discussions (e.g., global change, circular economy) and outreach activities will help an ECR to communicate, to critically place their work in a broader context and to judge the significance of their work as well as to develop new ideas about science. ECRs who are looking for their next post in academia should look for an environment of academic freedom where the advisor allows the ECR to develop competences, pursue their own ideas and projects, and build networks: all of which will improve their chances of pursing a successful career path of their choosing.

## Data Availability

Not applicable—No primary data was generated for this manuscript.

## Abbreviations

ECR: Early Career Researcher
NGO: Non-government organization
